# Biochemical properties of GH94 cellodextrin phosphorylase THA_1941 from a thermophilic eubacterium *Thermosipho africanus* TCF52B with cellobiose phosphorylase activity

**DOI:** 10.1038/s41598-017-05289-x

**Published:** 2017-07-07

**Authors:** Yuanyuan Wu, Guotao Mao, Haiyan Fan, Andong Song, Yi-Heng Percival Zhang, Hongge Chen

**Affiliations:** 1 College of Life Sciences, Henan Agricultural University, 95 Wenhua Road, Zhengzhou, 450002 China; 2 Biological Systems Engineering Department, Virginia Tech, 304 Seitz Hall, Blacksburg, Virginia 24061 USA

## Abstract

A hypothetic gene (THA_1941) encoding a putative cellobiose phosphorylase (CBP) from *Thermosipho africanus* TCF52B has very low amino acid identities (less than 12%) to all known GH94 enzymes. This gene was cloned and over-expressed in *Escherichia coli* BL21(DE3). The recombinant protein was hypothesized to be a CBP enzyme and it showed an optimum temperature of 75 °C and an optimum pH of 7.5. Beyond its CBP activity, this enzyme can use cellobiose and long-chain cellodextrins with a degree of polymerization of greater than two as a glucose acceptor, releasing phosphate from glucose 1-phosphate. The catalytic efficiencies (*k*_cat_/*K*_m_) indicated that cellotetraose and cellopentaose were the best substrates for the phosphorolytic and reverse synthetic reactions, respectively. These results suggested that this enzyme was the first enzyme having both cellodextrin and cellobiose phosphorylases activities. Because it preferred cellobiose and cellodextrins to glucose in the synthetic direction, it was categorized as a cellodextrin phosphorylase (CDP). Due to its unique ability of the reverse synthetic reaction, this enzyme could be a potential catalyst for the synthesis of various oligosaccharides. The speculative function of this CDP in the carbohydrate metabolism of *T*. *africanus* TCF52B was also discussed.

## Introduction

The β-d-glycoside phosphorylases, which act on the β-linked substrates such as cellobiose, cellodextrin, laminaribiose, N,N′-diacetylchitobiose, and cellobionic acid, are mainly classified into glycoside hydrolase family (GH) 94^1^. Among GH94 enzymes, cellobiose phosphorylase (CBP, EC 2.4.1.20) is the biggest member catalyzing the reversible phosphorolysis of cellobiose into α-d-glucose-l-phosphate (G-l-P) and d-glucose (Eq. 1). This enzyme exists in anaerobic bacteria, *e*.*g*., *Clostridium thermocellum* ^2^, *Ruminococcus albus* ^3^, *Cellvibrio gilvus* ^4^, *Clostridium stercorarium* ^5^, *Ruminococcus flavefaciens* ^6^, *Cellulomonas uda* ^7^, *Forms annosus* ^8^, *Thermotoga neapolitana* ^9^, and *Thermotoga maritima* ^10^. Cellobiose, an intermediate of enzymatic cellulose degradation, can be assimilated across the cellular membrane followed by intracellular phosphorolysis with CBP or by hydrolysis with β-glucosidase (βG). Bioenergetic studies demonstrated that intracellular phosphorolytic cleavage of cellobiose (or cellodextrins) was preferred to hydrolytic cleavage in cellulolytic anaerobic bacteria^11, 12^ because one ATP is saved per beta-1,4-glycosidic bond cleavage of glucose units of cellobiose and cellodextrins.

1 $${\rm{G}}\, \mbox{-} 1 \mbox{-} {\rm{P}}+{\rm{glucose}}={\rm{cellobiose}}+{{\rm{P}}}_{{\rm{i}}}$$

where G-1-P denotes glucose-1-phosphate, and *P*_*i*_ denotes inorganic phosphate.

Cellodextrin phosphorylase (CDP, EC 2.4.1.49) also belongs to the GH94 family, catalyzes the phosphorolysis of long-chain cellodextrins with a degree of polymerization (DP) of more than two and the synthesis of cellodextrin from short cellodextrins with a DP of at least two (Eq. 2).

2 $${\rm{G}}\, \mbox{-} 1 \mbox{-} {\rm{P}}+{{\rm{G}}}_{{\rm{n}}}={{\rm{G}}}_{{\rm{n}}+1}+{{\rm{P}}}_{{\rm{i}}}$$

where *G*_*n*_ denotes a β-glucan oligomer of length n (n ≥ 2), and *G*_*n*+*1*_ denotes a β-glucan oligomer of length n + 1. Although CBP and CDP belong to the same glycoside hydrolase family, they have different substrate specificities^13, 14^.

Laminaribiose phosphorylase (LBP, EC 2.4.1.31), N,N′-diacetylchitobiose phosphorylase (briefly noted chitobiose phosphorylase, ChBP, EC 2.4.1.280), and cellobionic acid phosphorylase (CBAP, EC 2.4.1.321) are relatively new members in GH94 family compared to CBP and CDP, catalyzing reversible phosphorolysis of their corresponding β-glucosidic substrates.

GH94 phosphorylases are involved in the intracellular catabolism of β-d-glycosides, enabling direct production of phosphorylated glucose without ATP consumption. Due to this energetic benefit, these enzymes could find broad uses in metabolic engineering, biocatalysis and *in vitro* synthetic biology. For example, CBPs have been introduced into non-cellulolytic ethanol-producing microbes, such as *Saccharomyces cerevisiae* and *Escherichia coli*, for intracellular phosphorolysis of cellobiose for enhanced bioenergetic levels^15–19^. Compared to cells employing a βG for intracellular cellobiose hydrolysis, Ha *et al*.^16^ demonstrate﻿﻿ that CBP-containing *S*. *cerevisiae* had more biomass synthesis and higher ethanol yields under strict anaerobic conditions and under acetate stress conditions. Shin *et al*.^17^ further shows that CBP-containing *E*. *coli* cells tolerated acetate more than BG-containing cells under both anaerobic and aerobic conditions. Another promising application of CBP is *in vitro* cascade biocatalysis. By utilization of its phosphorolytic ability, this enzyme can produce high-yield hydrogen from cellobiose and cellodextrin^20^ and from oligoxylosaccharides^21^. Also, by combining *C*. *thermocellum* CBP along with potato alpha-glucan phosphorylase, a fraction of cellulose can be converted to artificial starch^22^. By utilization of their reverse synthetic ability, CBP and CDP can be used to synthesize diverse disaccharides, oligosaccharides, and glycolipids^23–25^. Great potentials of CBP and CDP motivate us to discover their new species with more desirable properties, for example, high thermostability, high activity, broad substrate specificity and so on.

In this study, the open reading frame (THA_1941) encoding a putative CBP from *Thermosipho africanus* TCF52B was cloned and overexpressed in *E*. *coli* BL21(DE3). Its basic biochemical properties were characterized. This enzyme was the first enzyme with both cellodextrin and cellobiose phosphorylase activities.

## Results

### Discovery for a putative CBP from *T*. *africanus* TCF52B

By considering potential industrial applications and easy thermophilic protein purification expressed in a mesophilic host *E*. *coli*, putative thermostable CBP genes were searched from exploring genomic DNA database of thermophilic microorganisms. *T*. *africanus* TCF52B, which was isolated from a high-temperature oil reservoir in the North Sea with an optimal growth temperature of 70 °C, was sequenced and annotated in 2009^26^. Although the locus THA_1941 (protein_id ACJ76363.1) was annotated as a hypothetical protein in both KEGG and NCBI, there existed a region COG3459 in the sequence annotated as “cellobiose phosphorylase [carbohydrate transport and metabolism]”. So we speculated THA_1941 as a putative CBP. Also, signal peptide analysis predicted that the protein without a signal peptide was located intracellularly, in consistent with its intracellular function. However, sequence alignment with ClustalW showed that this putative 1,019 amino acid enzyme had a very low sequence identity to all characterized CBPs, as well as to characterized CDPs, LBPs, ChBPs, and CBAPs. Among them, it has the highest identity of 11.9% with *C*. *thermocellum* CBP (GenBank No.: ABN51514.1).

### Structure basis for the putative CBP’s enzymatic function

**Figure 1:**
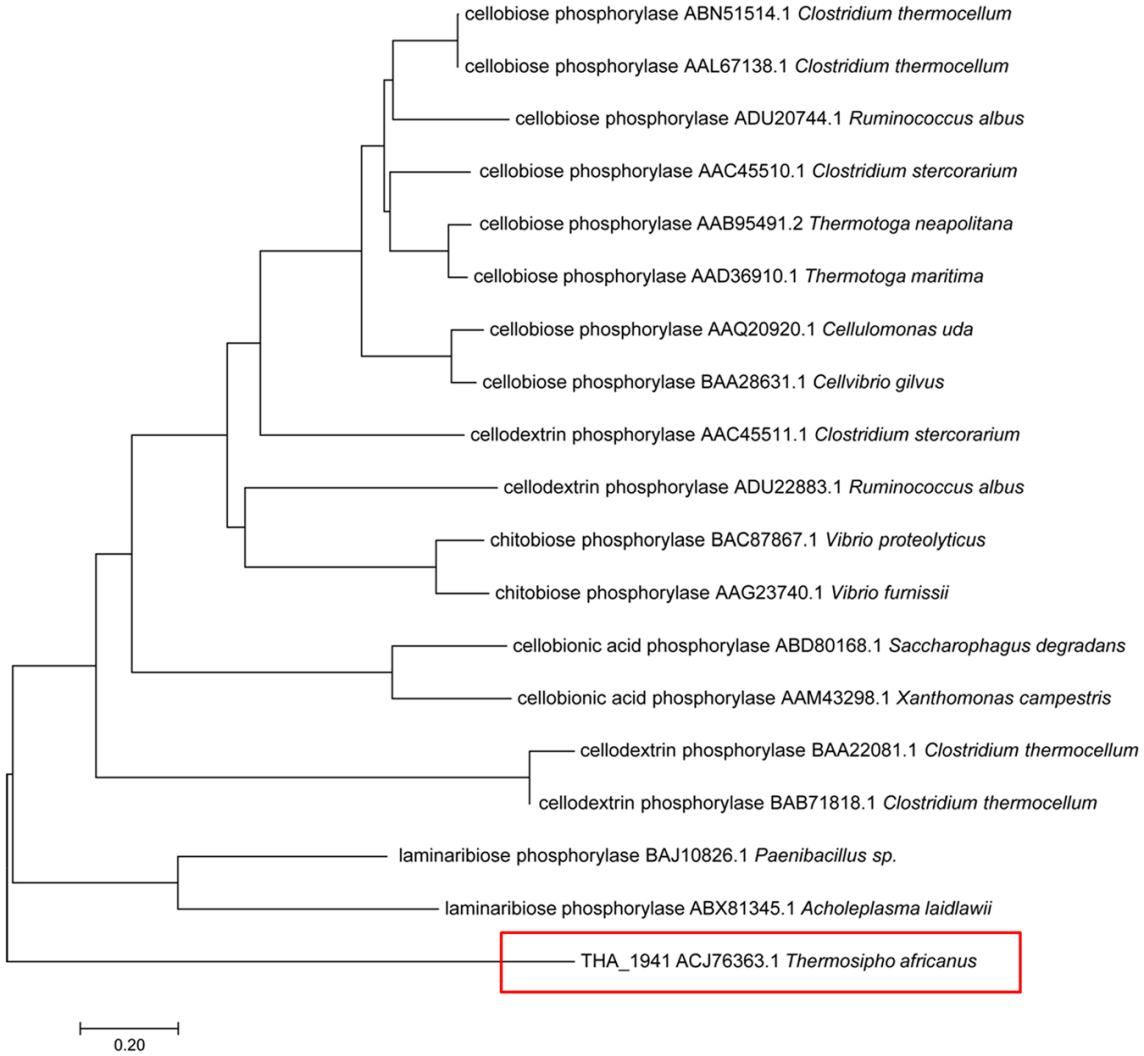
Phylogenetic relationship of THA_1941 with function-known GH94 enzymes. Phylogenetic tree was generated by Neighbour-Joining Method in MEGA software.

A phylogenetic analysis was conducted to examine the genetic relationship of THA_1941, the putative CBP with characterized GH94 phosphorylases, including CBPs, CDPs, LBPs, ChBPs, and CBAPs. The phylogenetic tree was generated by Neighbour-Joining Method based on amino acid sequences (Fig. 1). It was clear that all CBPs fell into one cluster, and ChBPs, CBAPs, and LBPs were all in their own cluster, while CDPs could go into the same cluster at the larger genetic distance with CBPs, ChBPs and CBAPs. Yet, THA_1941 separately located on an earliest diverging clade, indicating that the putative CBP had the greatest genetic distance with all others and therefore could not be categorized into any of the known GH94 enzymes.

**Figure 2:**
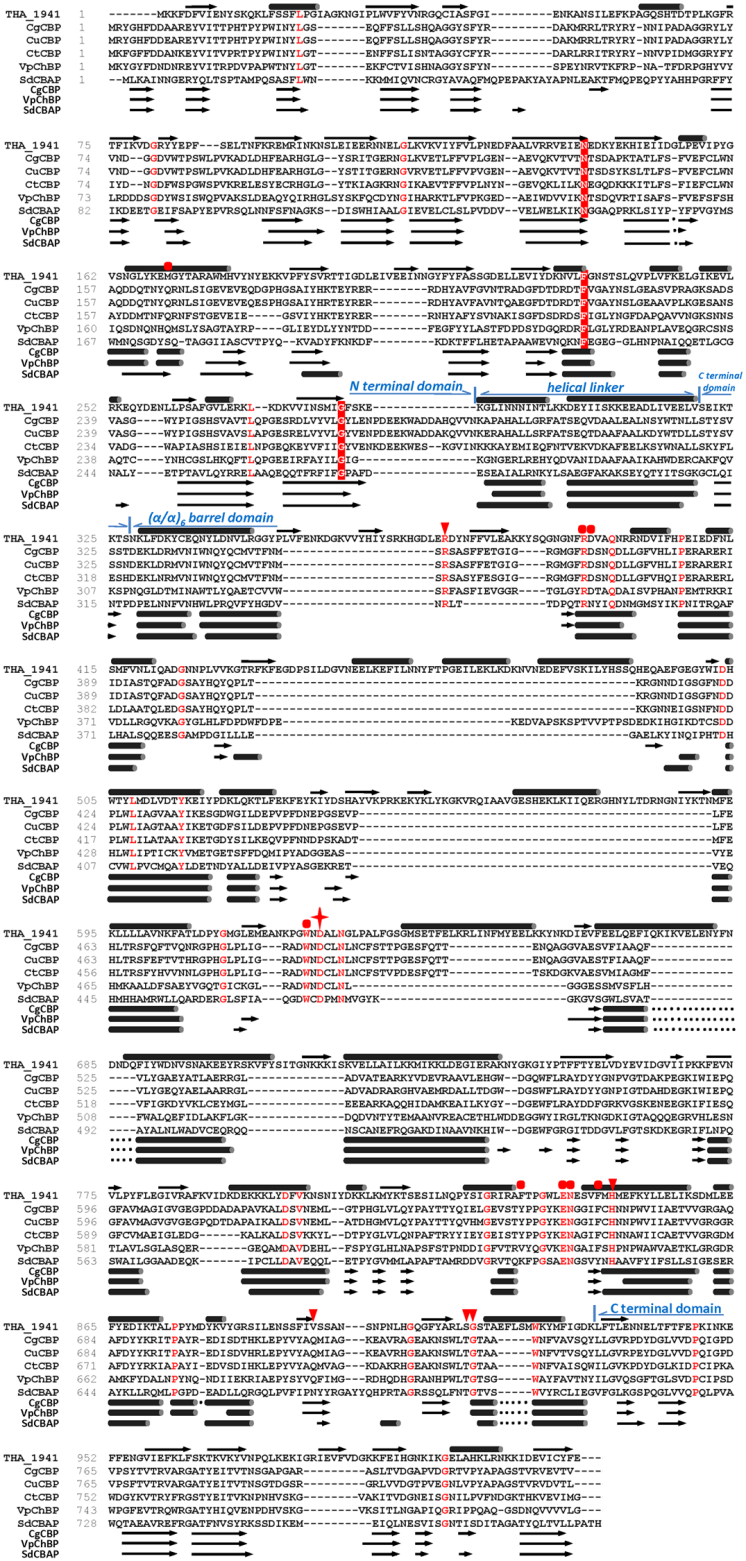
Amino acid sequence alignment of THA_1941 with structure-solved GH94 enzymes. Sequence alignment of THA_1941 with structure-solved CBPs from *Cellvibrio gilvus* (CgCBP, BAA28631.1), *Cellulomonas uda* (CuCBP, AAQ20920.1), and *Clostridium thermocellum* (CtCBP, AAL67138.1), ChBP from *Vibrio proteolyticus* (VpChBP, BAC87867.1), and CBAP from *Saccharophagus degradans* (SdCBAP, ABD80168.1) was performed using the program ClustalX2^42^ and formatted with BioEdit. Secondary structure was predicted with PSIPRED sever^43^ and the secondary structural elements are marked on the top. Secondary structure elements from CgCBP, VpChBP and SdCBAP are shown below the sequence alignment (the secondary structure of CgCBP represents that of CuCBP and CtCBP because of their high similarity). Arrows and columns represent the β strands and α helices, respectively. Conserved residues in the N terminal sandwich domain (*Glyco_trans_36*, PF06165, reclassficated into GH94) are highlighted with red rectangles. The catalytic residues are marked with red stars, while the phosphate binding sites and the sugar binding sites are marked with triangles and dots, respectively.

In order to find the conserved domains responsible for the putative CBP’s possible catalytic functions, those structure-solved enzymes in Fig. 1 were picked out for amino acid sequence alignment (i.e., *C*. *gilvus* CBP (CgCBP)^27^, *C*. *uda* CBP (CuCBP)^28^, *C*. *thermocellum* CBP (CtCBP)^29^, *V*. *proteolyticus* ChBP (VpChBP)^30^, and *S*. *degradans* CBAP (SdCBAP)^31^) (Fig. 2). The results showed that the secondary structural components of THA_1941 were almost the same as all the five enzymes despite low amino acid sequence identities. It can be divided into four distinct domains: N-terminal domain (1–287), a helical linker (288–319), (α/α)_6_-barrel domain (328–931), and C-terminal domain (320–327 and 932–1,019). The N-terminal β-sandwich domain was designated as *Glyco_trans_36* (PF06165) in Pfam database (later reclassified into GH94 family). In this domain THA_1941 had the conserved residues, such as Asn140, Phe230, Pro262 and Gly283, corresponding to Asn136, Phe217, Pro245 and Gly268 in CgCBP, respectively. The (α/α)_6_-barrel domain of THA_1941 was larger than that of the modular enzymes, owning to some insertions. Although low overall sequence identity in the (α/α)_6_-barrel domains, the potential catalytic residue Asp625 for THA_1941 (corresponding to Asp490 for CgCBP) near the center of (α/α)_6_-barrel was found based on the conserved sites in the modular enzymes. The potential phosphate binding sites in THA_1941 such as Arg373, His847, Ser913 and Gly914 (corresponding to Arg351, His666, Thr731 and Gly732 for CgCBP, respectively) and the potential sugar binding sites such as Arg393, Asp394, Trp623, Phe834, Glu840, Asn841, and Phe845 (corresponding to Arg367, Asp368, Trp488, Tyr653, Glu659, Asn660, and Phe664 for CgCBP, respectively), were also highly conserved.

Based on these structural similarities, THA_1941 was very likely to have the similar 3-D folding structure with the modular CBPs, ChBP, and CBAP and belong to GH94 family, having similar catalytic mechanism.

### Production, purification and CBP identification of THA_1941

**Figure 3:**
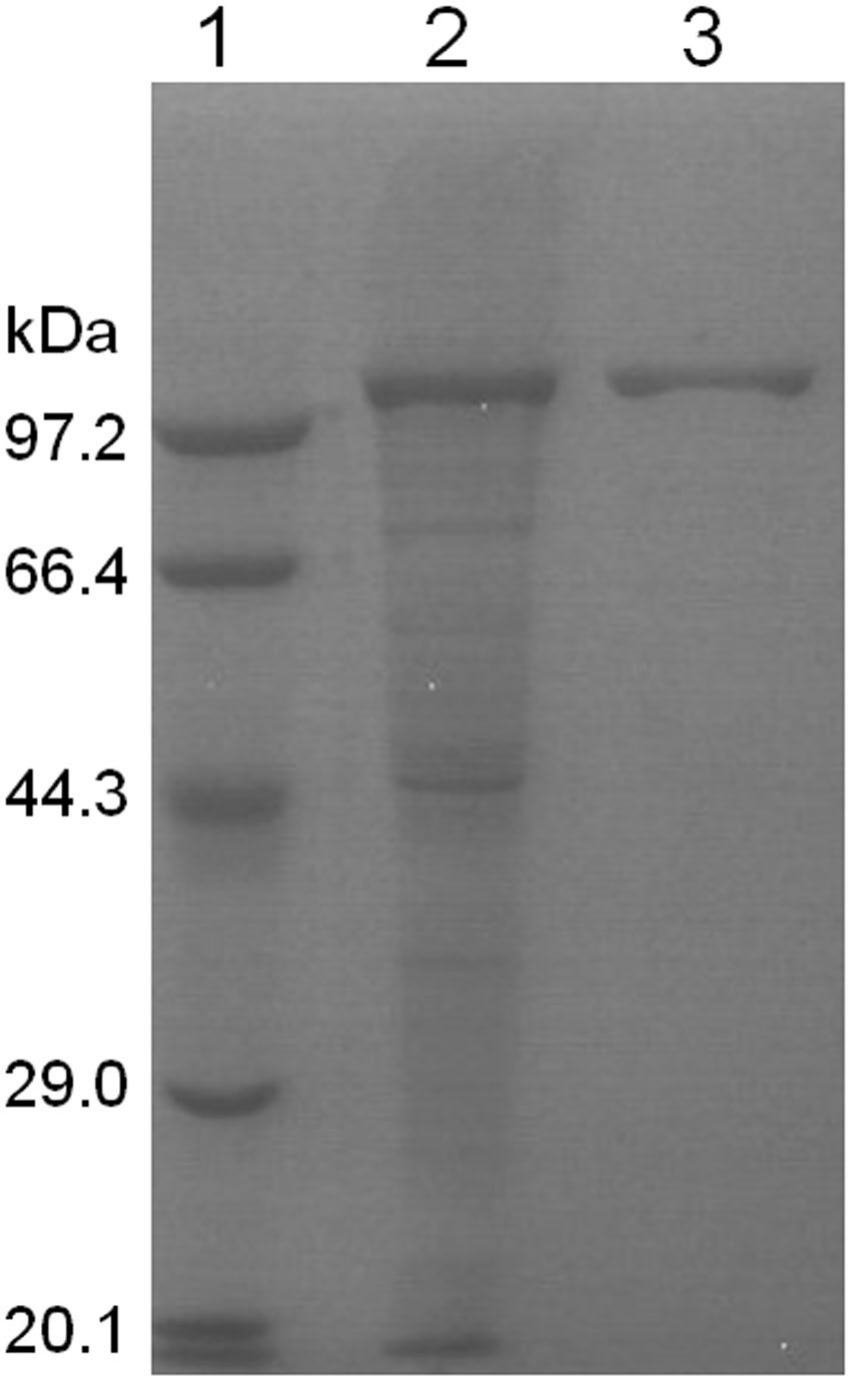
SDS-PAGE analysis of recombinant putative CBP. Lane 1, Molecular mass markers; Lane 2, Crude extract of recombinant *E*. *coli*; Lane 3, Purified THA_1941 protein.

**Figure 4:**
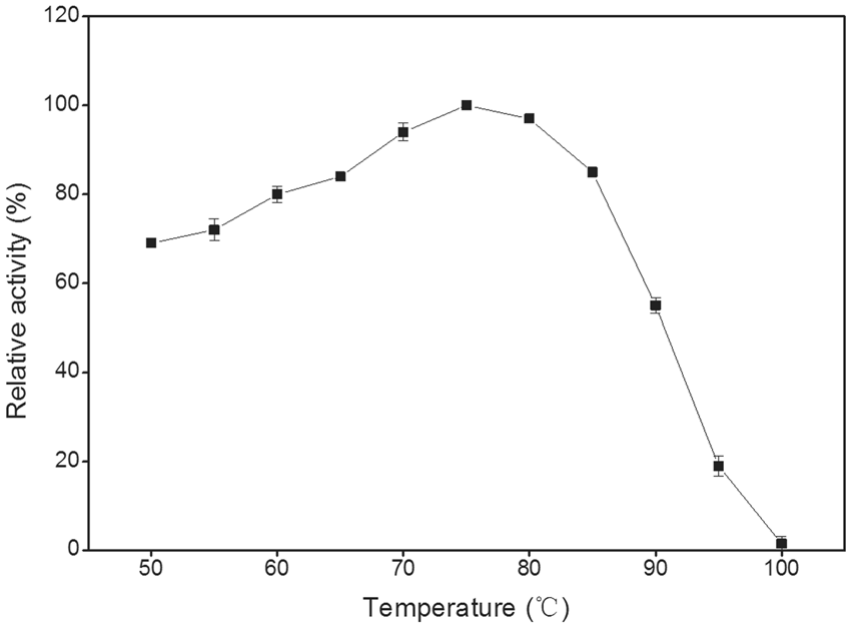
Effect of temperature on the activity of TaCBP.

**Figure 5:**
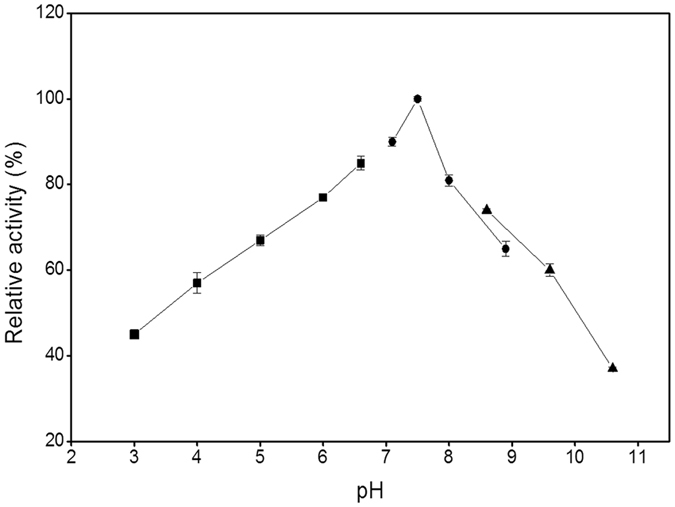
Effect of pH on the activity of TaCBP.

**Figure 6:**
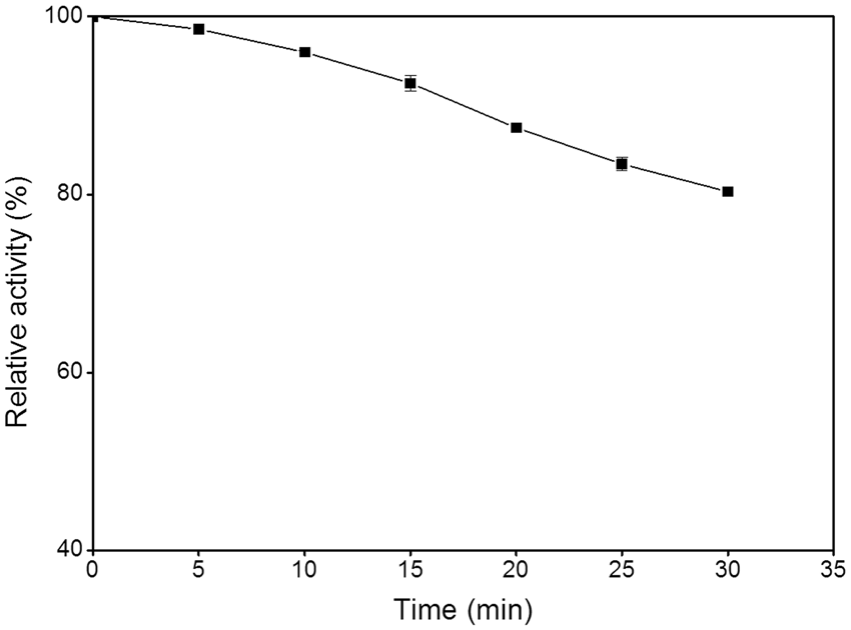
The stability of TaCBP incubated at 75 °C.

The recombinant THA_1941 was over-expressed by *E*. *coli* BL21(DE3) harboring the protein expression plasmid, and then was purified to homogeneity by Ni-chelating column chromatography followed by anion-exchange column chromatography. The purity of the protein was confirmed by SDS-PAGE analysis (Fig. 3). The molecular weight of the purified protein was estimated to be approximately 120 kDa, in agreement with its deduced amino acid sequence. The purified protein can release Pi from G-1-P with either d-glucose or d-xylose as a glucosyl acceptor. It also showed phosphorolytic activity towards cellobiose. Therefore, the hypothetical protein THA_1941 was validated to be a CBP enzyme (denoted TaCBP). TaCBP showed the highest activity at 75 °C (Fig. 4) and pH 7.5 (Fig. 5), and retained 80% of its initial activity after incubating at 75 °C and pH 7.5 for 30 min (Fig. 6), suggesting its good thermostability.

### Substrate specificity of TaCBP in the synthetic reaction

The synthetic reaction rates of TaCBP on 10 mM various sugars are presented in Table 1. TaCBP showed measurable activities to all nine monosaccharides, among which d-glucose had the highest rate of 1.86 µmol/min/mg, followed by d-glucosamine. As for three tested disaccharides, it was notable that both d-cellobiose and d-gentiobiose acted as glucosyl acceptors with 31.5- and 26.9-fold higher rates than that of d-glucose, respectively. Compared with these two disaccharides, the other disaccharide d-maltose showed a rather weak activity, furthermore, the possibility that the activity might be from contaminated d-glucose in the d-maltose was not yet excluded. Although the wide range of substrate specificity commonly existed in synthetic reaction of CBPs^3, 7, 10, 32^, there was no CBP reported to use cellobiose as a glucosyl acceptor. This result suggested that TaCBP was the first enzyme having both CBP and CDP activities.

**Table 1:** Substrate specificities of TaCBP in the synthetic reaction.

Substrate	Rate (µmol/min/mg)
d-Glucose	1.86 ± 0.06
d-Xylose	0.35 ± 0.01
d-Galactose	0.27 ± 0.02
d-Mannose	0.38 ± 0.01
d-Arabinose	0.22 ± 0.01
N-Acetyl-d-glucosamine	0.22 ± 0.01
d-Fructose	0.22 ± 0.01
d-Glucosamine	1.08 ± 0.04
2-Deoxy-d-glucose	0.85 ± 0.01
d-Maltose	0.16 ± 0.01
d-Cellobiose	58.61 ± 0.35
d-Gentiobiose	49.98 ± 0.28

Concentration of each substrate used was 10 mM.

### Identification of the CDP function

TaCBP’s activities towards various cellodextrins in the synthetic reaction and phophorolytic reaction were investigated in the presence of 5 mM different chain-length cellodextrins. Table 2 shows the activities of TaCBP on all cellodextrins from cellobiose to cellopentaose acted as glucosyl acceptors. It showed the highest activity of 109.3 µmol/min/mg on cellotetraose while remained 38.6% activity on cellobiose. In the phosphorolytic reaction, TaCBP showed measurable activities on all tested cellodextrins, although the reaction rates were much lower than those of synthetic reaction. Similar to the synthetic reaction, TaCBP exhibited the highest activity on cellotetraose. The fact that TaCBP can phosphorolyze both cellobiose and cellodextrins with a DP greater than two further validated that TaCBP had the catalytic function of a CDP enzyme.

**Table 2:** Substrate specificities of TaCBP on cellodextrins in the synthetic and phosphorolytic reactions.

Substrate	Synthetic direction		Phosphorolytic direction	
	Rate (µmol/min/mg)	Relative rate (%)	Rate (µmol/min/mg)	Relative rate (%)
Cellobiose	42.2 ± 0.20	38.6	0.069 ± 0.01	71.1
Cellotriose	98.1 ± 0.60	89.8	0.071 ± 0.01	73.2
Cellotetraose	109.3 ± 0.73	100.0	0.097 ± 0.01	100.0
Cellopentaose	69.2 ± 0.50	63.3	0.085 ± 0.02	87.6

Concentration of each cellodextrin used was 5 mM.

### Kinetic parameters

In the synthetic reaction, initial reaction rates were measured on varying concentrations of d-glucose, d-xylose, G-1-P, and cellodextrins and the kinetic parameters are summarized in Table 3. TaCBP showed a much higher catalytic efficiency on glucose (30.3 s^−1^) than on d-xylose (1.37 s^−1^). The *K*_m_ values for cellodextrins decreased with an increase in substrate chain length, suggesting TaCBP’s higher affinity on longer-chain substrates. The *k*_cat_ for cellotetraose was the highest among the cellodextrins tested, yet cellopentaose was the best substrate in terms of its *k*_cat_/*K*_m_.

**Table 3:** Kinetic parameters for the synthetic reaction catalyzed by TaCBP.

Substrate	*K*_m_ (mM)	*k*_cat_ (s^−1^)	*k*_cat_/*K*_m_ (s^−1^ mM^−1^)
d-Glucose^a^	14.3 ± 1.53	30.3 ± 2.20	2.12
d-Xylose^b^	25.7 ± 1.30	1.37 ± 0.13	0.05
G-1-P^a^	1.57 ± 0.20	7.61 ± 0.66	4.85
Cellobiose^c^	7.39 ± 0.75	361 ± 9.80	48.87
Cellotriose^c^	4.05 ± 0.31	603 ± 21.2	148.90
Cellotetraose^c^	3.7 ± 0.25	612 ± 18.8	165.46
Cellopentaose^c^	2.59 ± 0.22	527 ± 15.5	203.50

Concentrations used: ^a^0.5–10 mM; ^b^5–100 mM; ^c^0.5–5 mM.

The kinetic parameters to a series of cellodextrins for the phosphorolytic reaction were determined in the presence of 100 mM inorganic phosphate (Table 4). Similar with those for the synthetic reaction, the *K*_m_ values for cellodextrins decreased with an increase in substrate chain length. Cellotetraose was the best substrate for phosphorolysis in terms of the value of *k*_cat_/*K*_m_, consistent with the fact that TaCBP had the highest phosphorolytic activity towards cellotetraose.

**Table 4:** Kinetic parameters for the phosphorolytic reaction of TaCBP.

Substrate	*K*_m_ (mM)	*k*_cat_ (s^−1^)	*k*_cat_/*K*_m_ (s^−1^ mM^−1^)
Cellobiose	0.120 ± 0.03	0.23 ± 0.06	1.92
Cellotriose	0.094 ± 0.01	0.28 ± 0.05	2.98
Cellotetraose	0.062 ± 0.015	0.49 ± 0.10	7.91
Cellopentaose	0.060 ± 0.02	0.34 ± 0.11	5.67

Concentrations used: 0.02–1.0 mM.

## Discussion

We validated that the hypothetical protein THA_1941 from *T*. *africanus* TCF52B was an enzyme having both CDP and CBP activities. Comparing its two activities, though in phosphorolytic reaction they were in the similar level, in the synthetic reaction it showed remarkably higher specificity to cellodextins than to d-glucose and d-xylose, indicating its higher CDP activity than CBP activity. Therefore this enzyme was categorized as cellodextrin phosphorylase (EC 2.4.1.49) and designated as TaCDP despite of the initial name TaCBP.

Compared with properties of the known CBPs and CDPs (Table 5), TaCDP had the highest optimal temperature (75 °C) among known CDPs, representing its best thermostability. As for substrate specificity, no known CDP was reported to phosphorolyze cellobiose, neither to use monosaccharides such as d-glucose, d-xylose and d-glucose-derivatives as glucosyl acceptors in its reverse synthetic reaction. Different from known CDPs, TaCDP showed a wide range of substrate specificity in both reaction directions, making it a unique bifunctional enzyme with both CDP and CBP activities. It was noteworthy that TaCDP had much lower catalytic efficiency in the phosphorolytic reaction than in the reverse synthetic reaction, indicating that it had much greater preference for the synthetic reaction than for the phosphorolytic reaction. Moreover TaCDP’s catalytic efficiency in the synthetic reaction was shown to be the highest among the known CDPs as comparing their highest *k*_cat_ values for cellodextrins in synthetic reaction: the highest *k*_cat_ (612 s^−1^, 60 °C) of TaCDP was 13-fold and 38-fold higher than that of RaCDP (47.1 s^−1^, 37 °C)^33^ and CtCDP (16.2 s^−1^, 37 °C)^13^, respectively.

**Table 5:** Properties of chararcterized CBPs and CDPs from different strains.

Strains	Enzyme	MW (kDa)	pH optima	Temperature optima	Thermostability	Substrate specificity						Reference
						Synthetic reaction			Phosphorolytic reaction			
						Substrate	*K*_m_ (mM)	*k*_cat_ (s^−1^)	Substrate	*K*_m_ (mM)	*K*_cat_ (s^−1^)	
*Clostridium thermocellum* ATCC 27405	CBP	150	6.0–8.0	60 °C	<60 °C	α-d-Glucose-1-phosphate/d-Mannose/	8/170/	−/−/				32
						d-Xylose/d-Glucosamine/	40/10/	−/−/				
						2-Deoxy-d-glucose	250	—				
*Ruminococcus albus* NE1	CBP	187	6.2	50 °C	<40 °C	d-Glucose/d-Mannose/	1.46/23.5/	92.3/2.83/	Cellobiose	1.27	110	3
						2-Deoxy-d-glucose/d-Glucosamine/	60.2/13.3/	21.5/9.56/	Pi	0.427	—	
						d-Xylose/6-Deoxy-d-glucose/	25.5/9.8/	16.5/124/				
						1,5-Anhydro-d-glucitol/Gentiobiose	9.5/10.8	2.79/4.01				
*Clostridium stercorarium*	CBP	93	6.0-7.0	65 °C	50% residual activity after 20 h at 65°C				Cellobiose	6.2	1.1	4
*Thermotoga neapolitana*	CBP	93.5	5.0	85 °C	remained active for 2 h at 85°C				Cellobiose	1.42	26.3	9
*Clostridium thermocellum*	CBP	—	6.5(d-Xylose as acceptor)	—	—	6-Deoxy-glucose/d-Xylose/	9.2/35/	−/−/	Cellobiose	7.3	—	2
						2-Deoxy-d-glucose/d-Glucosamine/	73/9.5/	−/−/	Pi	2.9	—	
			7.5(2-Deoxy- glucose as acceptor)			d-Mannose/d-Arabinose/	85/240/	−/−/	d-Glucose	—	—	
						L-Fucose/α-d-Glucose-1-P	160/2.1	−/−				
*Thermotoga maritima* MSB8	CBP	150	6.2	80 °C	<70 °C	d-Glucose/d-Mannose/	0.69/67/	8/4.4/	Cellobiose	0.29	5.4	10
						d-Glucosamine/2-Deoxy-d-glucose/	5.7/47/	5.2/16/	Pi	0.15	—	
						6-Deoxy-d-glucose/d-Xylose/	4.1/14/	17/40/				
						Methyl-β-d-glucoside	135	6.5				
*Cellulomonas uda*	CBP	—	—	—	—	d-Glucose/β-d-Glucose/	2.26/4.7/	44.2/47.6/	d-Cellobiose	4.3	29.1	7
						2-Deoxy-d-glucose/	24.3/	11.6/	Pi	1.2	—	
						2-Deoxy-2-fluoro-d-glucose/	17.2/	8/				
						d-Mannose/d-Glucosamine/	27.3/10.8/	6.6/5.6/				
						3-Deoxy-d-glucose/	4/	0.1/				
						3-Deoxy-3-fluoro-d-glucose/	ND/	ND/				
						6-Deoxy-d-glucose/	10.2/	55.3/				
						6-Deoxy-6-fluoro-d-glucose/	7.4/	44.6/				
						d-Xylose/α-d-Glucose-1-P	15.9/2.15	8.7/−				
*Cellvibrio gilvus*	CBP	280	7.6	—	complete inactivation after 10 min at 60 °C				Cellobiose	1.25	—	4
									Pi	0.77	—	
*Prevotella ruminicola* (crude extracts)	CBP	—	—	—	—				Cellobiose	0.17	—	41
*Clostridium stercorarium*	CDP	91	6.0–7.0	65 °C	*t*_1/2 (60°C)_ = 30 h				Cellotriose	0.04	2.7	5
									Cellotetraose	0.05	6.9	
									Cellopentaose	0.17	6.6	
									Cellohexaose	0.19	6.1	
*Ruminococcus albus*	CDP	272	6.0	50 °C	<40 °C	Cellobiose/Cellotriose/	13.2/5.01/	47.1/43.7/	Cellobiose	ND	ND	33
						Cellopentaose/Cellohexaose/	2.73/3.22/	28.9/18.2/	Cellotriose	6.04	76.2	
						Sophorose/Laminaribiose/	343/119/	14.2/33.3/	Cellotetraose	4.16	92.8	
						Xylobiose/Mannobiose/	50.9/65/	15.4/41.3/	Cellopentaose	2.41	83.8	
						Cellobiitol//Cellotetraose	73.7//3.97	3.26//37.8	Cellohexaose	1.04	55.8	
*Clostridium thermocellum* YM4	CDP	210	7.5	60 °C	<60 °C	Glucose/Cellobiose/	ND/0.89/	ND/10.1/	Cellobiose	ND	ND	13
						Cellotriose/Cellotetraose	1.75/2.65	13.9/16.2	Cellotriose	0.81	4	
									Cellotetraose	0.82	3.2	
*Thermosipho africanus strain* TCF52B	CDP/CBP	120	7.5	75 °C	80% residual activity after 30 min at 75 °C	d-Glucose/d-Xylose/	14.3/25.7/	30.3/1.37/	Cellobiose	0.120	0.23	This work
						α-d-Glucose-1-P/Cellobiose/	1.57/7.39/	7.61/361/	Cellotriose	0.094	0.28	
						Cellotriose/	4.05/	603/	Cellotetraose	0.062	0.49	
						Cellotetraose/	3.7/	612/	Cellopentaose	0.060	0.34	
						Cellopentaose	2.59	527				

“−”: not tested; ND: not detected.

**Figure 7:**
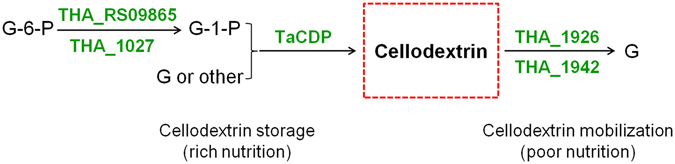
Presumed carbohydrate metabolism pathway with TaCDP involved in *Thermosipho africanus* TCF52B.

Though TaCDP’s enzymatic functions were characterized *in vitro*, it was difficult to investigate its biological function in *T*. *africanus* TCF52B without genetic modification tools. As suggested by Taylor II *et al*.^34^, both CBPs and CDPs were included in the class of “cellulase accessory enzymes”, which acted on cellulose oligosaccharides, the products of cellulases *i*.*e*. β-1,4-endoglucanases and/or cellobiohydrolases. By searching the genomic sequence of *T*. *africanus* TCF52B, only 2 ORFs (THA_83 and THA_328) were found to be possible endoglucanases related to cellulose degradation, yet both of them have no putative signal peptides. Hence, we presumed that *T*. *africanus* TCF52B was unlikely to be a lignocellulose-degrading bacterium. Considering the fact that TaCDP’s phosphorolysis efficiency for cellodextrins was much lower than its synthesis efficiency, we then speculated that TaCDP might be responsible for the cellodextrins formation other than for cellodextrins degradation, where cellodextrins formed by TaCDP might be intracellular energy reserve material, like poly-β-hydroxybutyrate accumulated in many bacteria^35^ and glycogen accumulated by *C*. *cellulolyticum* ^36^. A possible carbohydrate metabolism pathway was constructed to illustrate the role of involved TaCDP (Fig. 7). Here, when *T*. *africanus* TCF52B grows in rich nutrition conditions, cytoplasmic G-6-P will be converted to G-1-P by phosphoglucomutase (locus THA_RS09865 and THA_1027) and G-1-P then transfer its glucose unit to d-glucose or other monosaccharides by TaCDP, forming gradually longer cellodextrin. When this bacterium grows in poor nutrition conditions, accumulated cellodextrin will be hydrolyzed by intracellular β-glucosidase (locus THA_1926 and THA_1942). This scheme for synthesizing energy reserves uses ATP-derived energy more efficiently than that of synthesizing glycogen, as only one ATP is consumed per glucose unit addition while two ATPs in glycogen synthesis. Actually, we did not find any putative UDP/ADP-glucose pyrophosphorylases in *T*. *africanus* TCF52B genome, enzymes responsible for activated glucosyl donor formation for the glycogen synthesis, which may increase the possibility of our assumption on TaCDP’s function.

Phosphorolytic enzymes like CBPs and CDPs had advantages in oligosaccharides synthesis over chemical catalysts because they have both stereoselectivity and regiospecificity^37, 38^. TaCDP had high synthetic activities towards monosacchrides, disaccharides, and long-chain oligosaccharids, making it a valuable biocatalyst for cost-effective enzymatic synthesis of various oligosaccharides.

## Methods

### Cloning and expression of *T*. *africanus* THA_1941

The DNA sequence of THA_1941 can be found in KEGG database. The gene was amplified using the polymerase chain reaction (PCR) from the *T*. *africanus* TCF52B genomic DNA using 5′-CCTAG CTAGC ATGAA AAAAT TTGAC TTTGT G-3′ and 5′-CCGCT CGAGT TCAAA ATAAC ATATA ACTTC GTC-3′ as the forward and reverse primers, respectively (*Nhe*I and *Xho*I restriction sites underlined, respectively). The PCR product was digested with *Nhe*I and *Xho*I prior to insertion into pET21a(+). The ligation product was transformed into *E*. *coli* DH5α competent cells and the plasmid was verified by DNA sequencing service provided at Genewiz Inc. China. The plasmid was then transformed into *E*. *coli* BL21(DE3) competent cells. A single colony was picked to grow in Luria-Bertani (LB) medium supplemented with 50 μg/ml ampicillin. The culture was grown at 37 °C and 220 rpm until an absorbency of 600 nm reached 0.6–0.8. Expression of the protein was induced with the addition of isopropyl β-d-1-thiogalactopyranoside (IPTG) to a final concentration of 1 mM and the culture temperature was decreased to 25 °C for six hours. The cells were harvested by centrifugation at 3,800 × g for 10 min at 4 °C.

### Enzyme purification

The cell pellets were re-suspended in 20 mM Tris-HCl buffer (pH 7.0) with 500 mM NaCl (pH8.0) followed by sonication. The cell lysate was centrifuged at 10,000 × g at 4 °C for 20 min and the supernatant was applied to a nickel-charged resin column (Takara, Shiga, Japan). The column was washed with gradual washing containing 20–50–200–500 mM imidazole. The fraction eluted with 200 mM imidazole was collected and dialyzed against 20 mM Tris-HCl buffer (pH 7.4). After centrifugation the enzyme solution was loaded onto a pre-equilibrated HiTrap Q HP column (GE Healthcare) and the elution was performed with a linear gradient of NaCl from 100 to 500 mM. Under these conditions, most target protein was in the fractions of 200 mM NaCl eluate. The purity of each fraction was assessed by SDS-PAGE^39^. Only those fractions showing a single band were pooled and the concentration of the resulting purified sample was determined to be 1.40 mg/ml using the method of Bradford with bovine serum albumin as a standard^40^.

### Enzyme assays

In the synthetic reaction, CBP activity was assayed by measuring the amount of P_i_ liberated from G-1-P^32^. d-Glucose was routinely used as an acceptor of glucosyl, unless otherwise noted. A reaction mixture of 200 μl contained 50 μl appropriately diluted enzyme, 40 mM G-1-P, 1 mM MgCl_2_, 10 mM dithiothreitol (DTT), 20 mM d-glucose, and 50 mM Tris-HCl buffer (pH 7.5). The mixture was incubated for 15 min at 60 °C and then the reaction was terminated by the addition of 2 ml of the molybdate reagent containing 15 mM ammonium molybdate, 100 mM zinc acetate [pH 5.0]), and 500 μl of ascorbic acid reagent (10% [wt/vol], pH 5.0) was then added to the mixture. This mixture was incubated at 30 °C for 15 min, and the absorbance was measured at 850 nm. One unit of CBP in synthetic reaction was defined as the amount of enzyme that produced 1 μmole of phosphate per min.

The phosphorolytic activity of TaCBP was assayed by measuring the formation of G-1-P from cellobiose (or cellodextrins when stated). A reaction mixture of 40 μl containing 5 mM cellobiose or cellodextrins, 100 mM sodium phosphate buffer (pH 7.5), and appropriate amount of enzyme was incubated for 15 min at 60 °C. The reaction was stopped by boiling for 10 min, and the amount of G-1-P produced was determined by a coupled enzyme assay measuring the appearance of NADPH at 340 nm. The assay mixture contained phosphoglucomutase (4.0 U/ml), glucose-6-phosphate dehydrogenase (2.0 U/ml), where the two enzymes were purchased from Sigma, 3 mM NADP^+^, and 5 μM glucose 1,6-bisphosphate (Sigma) in 80 mM triethanolamine buffer (pH 7.5) containing 4.4 mM MgCl_2_. One unit of phosphorolytic activity was defined as the release of one μmole of G-1-P per min.

### Optimum pH, optimum temperature, and thermal stability

Determinations in this section were all based on the synthetic reaction. The optimum pH was investigated by measuring the enzyme activity as described above at various pH values (citric acid-sodium citrate buffers for pH 3.0–6.6, Tris-HCl buffers for pH 7.1–8.9, and glycine-NaOH buffers for pH 8.6–10.6). The optimum temperature was measured over a temperature range of 50–100 °C at pH 7.5. To evaluate thermostability, the enzyme (0.092 mg/ml) was incubated at 75 °C and pH 7.5 for different time periods (5–30 min) and the residual enzyme activity was measured. All assays were performed in triplicates.

### Substrate specificity

The substrate specificity for the synthetic direction was investigated by measuring initial velocities (μmol/min/mg) for 10 mM various sugars with 40 mM G-1-P at 60 °C. The final concentration of enzyme used for each sugar was adjusted properly to make sure the P_i_ amount released was suitable for accurate determination. Thus the final enzyme concentrations were 0.67 µg/ml and 2 µg/ml for d-cellobiose and d-gentiobiose, respectively, while 20 µg/ml of enzyme was used for d-glucose, d-maltose, and 2-deoxy-d-glucose, and 100 µg/ml for all other sugars.

The substrate specificity for the phosphorolytic direction was investigated by measuring the initial velocities for 5 mM different cellodextrins at 60 °C with 87.5 µg/ml of the enzyme. The other conditions were the same as for the phosphorolytic activity assay mentioned earlier.

### Kinetic analyses

To determine the kinetic parameters for the synthetic reaction, 200 µl of reaction system was used and the final concentrations of the enzyme used for d-glucose, d-xylose, and cellodextrins were 20, 100, and 0.67 µg/ml, respectively. The initial reaction rates at varying concentrations of d-glucose (0.5–10 mM), d-xylose (5–100 mM), or cellodextrins (0.5–5 mM) with 40 mM of a fixed concentration of G-1-P were determined. While determining the kinetic parameters to G-1-P, the reaction mixture contained 20 mM of a fixed concentration of d-glucose and G-1-P (0.5–10 mM) with 20 µg/ml of the enzyme. To determine the kinetic parameters for the phosphorolytic reaction to cellodextrins, 40 µl of reaction system and 100 µg/ml of the enzyme concentration were used. The initial reaction rates at varying concentrations of cellodextrins with 100 mM sodium phosphate buffer (pH 7.5) were determined. Both the synthetic reaction and the phosphorolytic reaction were incubated at 60 °C for 10 min. Each result was an average of at least three repetitions. *K*_m_ and *k*_*cat*_ values were calculated based on the Hanes-Woolf plots.
